# Adherence to the face-down positioning after vitrectomy and gas tamponade: a time series analysis

**DOI:** 10.1186/s13104-018-3257-1

**Published:** 2018-02-20

**Authors:** Keita Suzuki, Yoshiaki Shimada, Yui Seno, Tadashi Mizuguchi, Atsuhiro Tanikawa, Masayuki Horiguchi

**Affiliations:** Department of Ophthalmology, Fujita Health University School of Medicine, Fujita Health University Hospital, 1-98 Dengakugakubo, Kutsukake-cho, Toyoake, Aichi 470-1192 Japan

**Keywords:** Adherence, Face-down positioning, Gas tamponade, Macular hole, Retinal detachment, Vitrectomy

## Abstract

**Objective:**

To determine the adherence to the face-down positioning (FDP) in 296 patients who had undergone vitrectomy and gas tamponade.

**Results:**

We studied 119 female and 177 male patients who had undergone primary vitrectomy and gas tamponade for a macular hole (MH) or for rhegmatogenous retinal detachments (RRDs). Adherence was assessed and recorded four times per day for 3 days postsurgery. The mean ± standard deviation adherence rate was 88.3 ± 11.7 (range 50.0–100.0). Female patients (90.8 ± 10.0) had significantly better adherence than male patients (86.7 ± 13.3; *P* < 0.02, *U* test). The adherence was significantly better after MH surgery (90.8 ± 11.7) than after RRD surgery (87.5 ± 12.5; *P* < 0.02). There were diurnal variations in adherence, being highest in the evening and significantly lower at midnight, and we evidenced a decline in adherence over time, with it being significantly low on the last follow-up day. Adherence to the FDP varied considerably among patients. Adherence was higher in female than in male patients, and higher in patients with MH than in those with RRD. We found patients age had no effect on adherence. Adherence also varied with time, being worst at midnight and declining over time.

**Electronic supplementary material:**

The online version of this article (10.1186/s13104-018-3257-1) contains supplementary material, which is available to authorized users.

## Introduction

Face-down positioning (FDP) is recommended after vitrectomy and gas tamponade for rhegmatogenous retinal detachments (RRDs) [1, 2] or for macular hole (MH) surgery [1–33]. But FDP is inconvenient and not readily tolerated; thus, the optimal method and duration of FDP have been debated for many years, especially after MH surgery [2, 4–33]. Shortening the duration of FDP [2, 4, 7, 10, 12, 13, 16, 18, 19, 23, 30] and the use of alternate positions, such as avoidance of the supine or face-up positions [8–15, 17, 20, 21, 23–26, 29, 32, 33], has been proposed after comparisons with strict FDP [7, 9–14, 22–26, 29, 33]. However, in these studies the patients’ actual positions were not assessed, and thus the researchers did not actually test the positions adopted by the patients [22]. In other words, although the same advice was given to all the patients, probably some patients complied strictly, whereas others did not.

A mechanical sensory device mounted on the patient’s head to automatically monitor the head position [6, 22, 32] or the nursing records of direct observation of patient’s position [34, 35] can both be used to obtain an index of adherence. A study on 127 patients who had undergone primary vitrectomy for RRD revealed a mean adherence rate during 3 postoperative days of 85.0%, with considerable variation among patients and better adherence by the female patients, but without associations to the outcomes [34]. And, a survey of 69 patients with MH found a mean adherence rate of 88.3%, and failure of the MH closure was observed in the one patient who showed the poorest adherence (33.3%) [35]. Still, the reality of patients adherence has been largely unexplored. In this study, we assessed the influence of gender, age, and causes of surgery, and daily and day-by-day variations on the adherence.

## Main text

### Methods

#### Methodology and subjects

We retrospectively examined the nursing records of all hospitalized patients who had undergone primary vitrectomy and gas tamponade and had stayed at least 3 postoperative days at Fujita Health University Hospital (Toyoake, Japan) between April 2012 and March 2014. In our nation, staying in hospital for several days to undergo retinal surgery is common. Each patient was advised to adhere to the FDP indication after the surgery. A total of 296 patients (119 females and 177 males) were included; of these, 204 had had RRD and 92 had had MH. The nursing records included direct observations 4 times a day regarding the patients adherence to the FDP indication.

#### Surgery

The patients who signed the consent forms for surgery received instructions for FDP. Experienced surgeons performed the pars plana vitrectomies. Prophylactic phacoemulsifications and intraocular lens implantations were performed in 243 patients. Of the other 53 patients, one had an aphakic eye and 18 had intraocular lens implants. Each patient also underwent gas tamponade with either 20% sulfur hexafluoride (SF_6_) or 15% perfluoropropane (C_3_F_8_). All patients were advised to maintain the FDP post-surgery.

#### Nursing records

Since around 2001, our nurses have directly observed and recorded the patients positions on the nursing records using a handheld terminal during the four routine ward rounds per day. The Information regarding gas tamponades on the patients was noted in the hospital chart. The instruction for the patients included the possibility of adopting either a prone of sitting position during the FDP. The relevant data were then exported and stored in a digital hospital chart. The nurses instructed the patients to continually maintain the FDP. If the patients were seen sleeping in a position other than the FDP, a nurse would wake them up and asked them to maintain the FDP.

#### Adherence rate

**Fig. 1:**
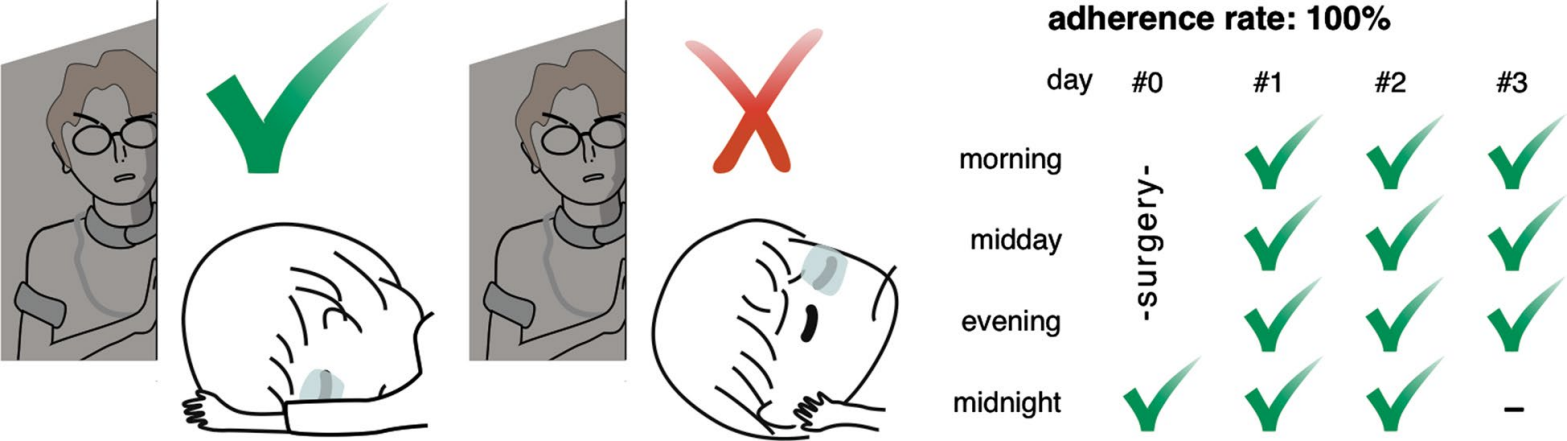
FDP adherence estimation: Patients were checked to determine if they adhered to the FDP (top panel) four times per day as follows: at midnight (24:00 h), in the morning (6:00 h), at midday (12:00 h), and in the evening (18:00 h), starting from midnight on the day of the surgery and continuing fro up to three consecutive days

To calculate the patients’ adherence to FDP, the position of each patient was checked four times per day: at midnight (24:00 h), in the morning (6:00 h), at midday (12:00 h), and in the evening (18:00 h). Patient monitoring began at 24:00 h on the day of the surgery. Although the nurses continued these examinations until the gas disappeared or the patient was discharged, we included only the first three consecutive days postsurgery in our initial study [34, 35]. Therefore, a total of 12 observations were recorded for each patient (Fig. 1). The adherence rate was obtained by dividing the number of times the patients were found in the FDP by 12 and multiplying by 100. For example, if a patient was found in a position other than the FDP in two out of 12 observations, the adherence rate was (12–2)/12, 83.3%.

### Results

#### Outline and gender/disease comparison

Table 1 shows the demographic data of the patients. The mean ± standard deviation of adherence rate [%] in all patients was 88.3 ± 11.7 (range 50.0–100.0).

**Table 1:** Subjects and adherence rate

All subjects		Sex		Disease	
		Female	Male	RRD	MH
n [cases]	296	119	177	204	92
		RRD: 63	RRD: 141		
		MH: 56	MH: 36		
Eye [cases, right/left]	151/145	63/56	88/89	99/105	52/40
Age [years, mean ± SD]	59.0 ± 11.4	61.5 ± 10.5	57.4 ± 11.7	56.2 ± 11.2	65.3 ± 9.3
Adherence rate [%]					
[Mean ± SD]	88.3 ± 11.7	90.8 ± 10.0	86.7 ± 13.3	87.5 ± 12.5	90.8 ± 11.7
A perfect 100% [cases]	105 (35.5%)	47 (39.5%)	58 (32.8%)	64 (31.4%)	41 (44.6%)
Less than 66.7% [cases]	13 (4.4%)	2 (4.2%)	11 (6.2%)	9 (4.4%)	4 (4.3%)

**Fig. 2:**
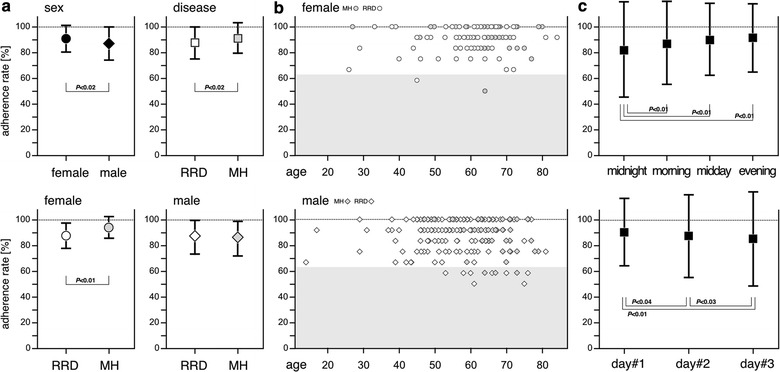
Adherence rates. **a** Mean adherence rates: the mean adherence rates were compared between female and male patients (top left panel) and between patients with RRD and those with MH (top right panel); single-gender comparisons of diseases were performed among female (bottom left) and male (bottom right). *P* values were obtained using the Mann–Whitney *U* test. **b** Distribution of adherence rates: function of patient age in female (top panel) and male (bottom panel) patients. **c** Summarized plots for daily (upper panel) and day-by-day (lower panel) variations: *P* values were obtained using Cochran the Q test with Bonferroni correction

The adherence rates are plotted in Fig. 2. The mean adherence rate was significantly better in female (90.8 ± 10.0) than in male (86.7 ± 13.3; *P* < 0.02, Mann–Whitney *U* test; Fig. 2a, top left panel), and significantly worse in patients with RRD (87.5 ± 12.5) than in those with MH (90.8 ± 11.7; *P* < 0.02, Mann–Whitney *U* test; Fig. 2a top right panel). We also performed single-sex comparisons between RRD and MH. The mean adherence rate was significantly worse in female patients with RRD (89.2 ± 10.0) than in female patients with MH (94.2 ± 9.2; *P* < 0.01, Mann–Whitney *U* test; Fig. 2a bottom left panel). And, it was slightly better in male patients with RRD (87.5 ± 13.3) than in those with MH (85.8 ± 13.3), but the difference was not statistically significant (*P* > 0.6, Mann–Whitney *U* test; Fig. 2a, bottom right panel).

#### Influence of patient age

Figure 2b shows the distribution of adherence rates as a function of patient age. No significant correlation was observed between adherence rate and patient age regardless of gender (females, *P* > 0.13; males, *P* > 0.95; all patients, *P* > 0.27; Pearson’s correlation coefficient test).

#### Time series analysis

Daily and day-by-day variations in adherence are plotted in Fig. 2c. The exact adherence rates after 12 observational points are presented in an Additional file 1. The adherence rate was highest in the evening of day 1 (95.3 ± 21.3) and lowest (80.7 ± 39.5) at midnight on day 3. Among the observational time points, statistically significant differences were observed in many particular comparisons (Cochran *Q* test with Bonferroni correction). For simplicity, these differences were summarized as occurring daily (Fig. 2c, top panel) and on a day-by-day basis (Fig. 2c bottom panel) variations.

Regarding day-by-day variation in adherence, the adherence rate at midnight (83.4 ± 37.2) was significantly lower than that in the morning (88.4 ± 32.0; *P* < 0.01), at midday (90.7 ± 29.1; *P* < 0.01) or in the evening (90.7 ± 29.1; *P* < 0.01, Cochran *Q* test with Bonferroni correction).

Regarding daily variations, the adherence rate on day 1 (91.7 ± 27.6) was significantly higher than that on day 2 (88.6 ± 31.8; *P* < 0.04) and on day 3 (85.3 ± 35.4; *P* < 0.01). The adherence rate on day 2 was also significantly higher than that on day 3 (*P* < 0.03, Cochran *Q* test with Bonferroni correction).

### Discussion

It should be noted that the patients surveyed in this study were kept in the hospital under constant observation. If they had stayed home after the surgery, the adherence would probably have been worse [34, 35]. As shown in our previous studies [34, 35], adherence considerably varied among patients and was higher in female than in male patients. Interestingly, the patients’ age had little effect on the adherence rate [34, 35]. It has been suggested that patients with MH have higher adherence than those with RRD [35]. On this study a greater proportion of females were being treated for MH than for RRD, but our results agreed with the suggested better adherence for MH patients. These findings may be attributed to the differences in urgency between treatments for MH and RRD [35]. While many patients with RRD undergo surgery on the first day of their hospital visit and have little time to prepare, the patients with MH have an elective procedure with sufficient time to plan ahead and prepare for the FDP. The self-decision and preparation time prior to the surgery may improve the patient adherence in MH cases [35].

We found the patient adherence varied with time. The worst adherence was at midnight, which may be attributable to deep sleep, as implied in our previous studies [34, 35] and in other studies using a head-mount monitoring device [6, 32]. The effort required to maintain the FDP when asleep may be different from that required to maintain the FDP when awake [35]. Even on day 1, immediately postsurgery, approximately one-seventh of the patients failed to maintain the position.

Whether the decline in adherence over time postsurgery is due to relaxed persona goal standards caused by the passage of time remains unclear. In our study, we followed patients for only 3 days postsurgery. If follow-up had been conducted for longer than a week, the attenuation of adherence might have been more intense. We have suggested that attempts to shorten the duration of FDP [2, 4, 7, 10, 12, 13, 16, 18, 19, 23, 30] to treat MH may result in an increase in practical adherence.

In the treatment of MH, the optimal manner and duration of FDP to optimize patient recovery have been debated [2, 4–33]. Although each protocol assumes that the patients will follow the given advice, some patients are noncompliant and may negatively impact the effectiveness of the surgery by being the sources of negative outcomes [35].

In conclusion, surgeons should not expect patients to always comply with the advice provided regarding FDP, because adherence varies among patients. We found adherence was higher in female than that in male patients, and higher among patients with MH than in those with RRD; however, patient age had little effect on adherence. Adherence also varied with time, being worst at midnight and declining over time.

### Limitations

The data for our study were retrospectively obtained from the nursing records but were used to evaluate many patients. Our observational assessment of adherence was based on a sampling frequency of only four times per day. While a mechanical sensory device mounted on the patient’s head has provided reliable data in previous studies [6, 22, 32] we believe the mounted device to monitor the head position could increase the strain on the patients.

## Additional file

**Additional file 1.** The adherence rates at 12 observational points.

## Abbreviations

C_3_F_8_: octafluoropropane
FDP: face-down positioning
MH: macular hole
RRD: rhegmatogenous retinal detachment
SF_6_: sulfur hexafluoride
