# Supplementary figures and images for: Adherence to the face-down positioning after vitrectomy and gas tamponade: a time series analysis
Source: BMC Res Notes. 2018 Feb 20;11:142. doi: 10.1186/s13104-018-3257-1 (PMC5819221; doi:10.1186/s13104-018-3257-1)

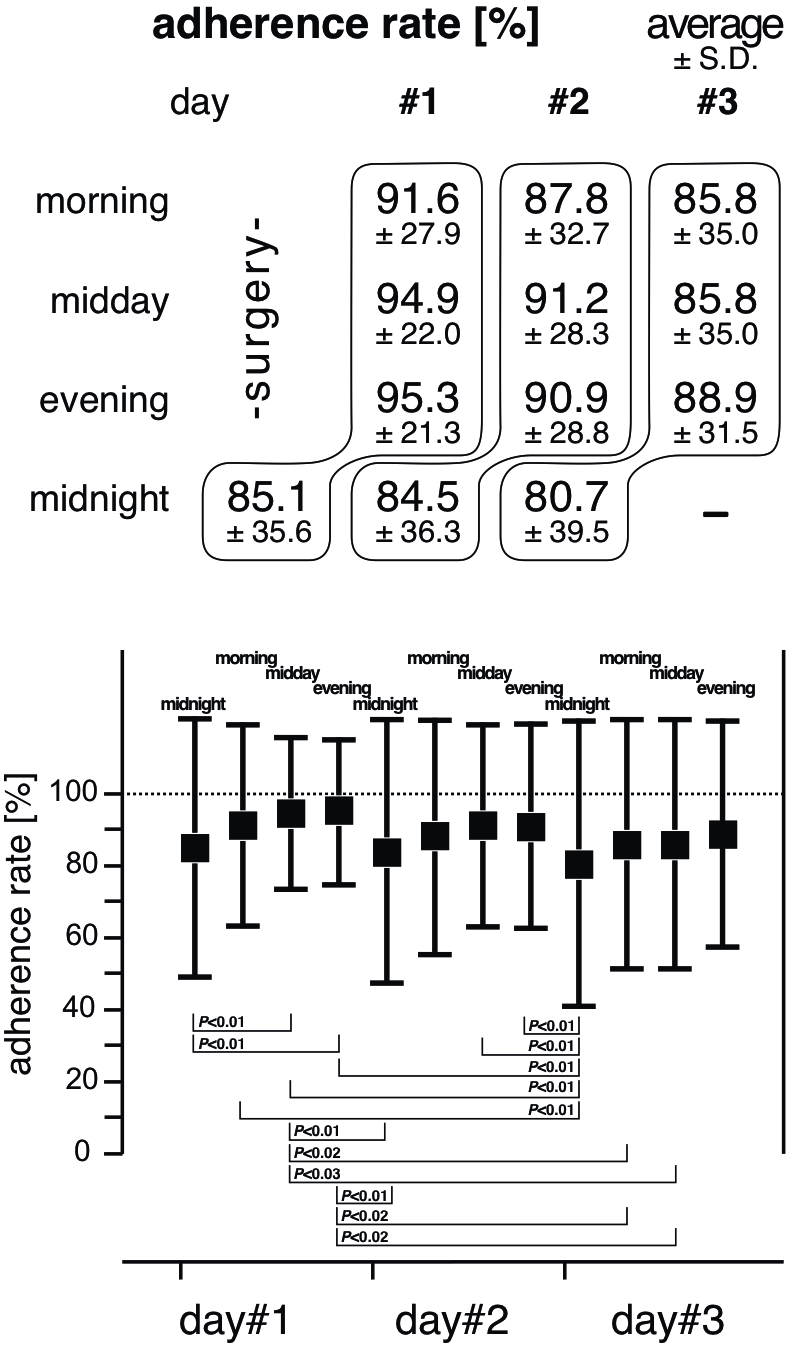

Supplement: Supplementary file 1 — Additional file 1. The adherence rates at 12 observational points. [file 13104_2018_3257_MOESM1_ESM.tiff]
